# *Borrelia burgdorferi* Infection and Cutaneous Lyme Disease, Mexico

**DOI:** 10.3201/eid1310.060630

**Published:** 2007-10

**Authors:** Guadalupe Gordillo-Pérez, Javier Torres, Fortino Solórzano-Santos, Sylvie de Martino, Dan Lipsker, Edmundo Velázquez, Guillermo Ramon, Muñoz Onofre, Benoit Jaulhac

**Affiliations:** * Instituto Mexicano del Seguro Social, Mexico City, Mexico; † Hôpitaux Universitaires, Strasbourg, France

**Keywords:** Lyme borreliosis, lymphocytoma, erythema migrans, Borrelia burgdorferi, dispatch

## Abstract

Four patients who had received tick bites while visiting forests in Mexico had skin lesions that met the case definition of erythema migrans, or borrelial lymphocytoma. Clinical diagnosis was supported with histologic, serologic, and molecular tests. This study suggests the *Borrelia burgdorferi* infection is in Mexico.

Lyme disease is the most frequently reported vectorborne infectious disease in the United States and Europe (*1*,*2*). Studies have suggested that *Borrelia burgdorferi* infection might be endemic to Mexico (*3*,*4*). We searched for histologic, immunologic, and molecular evidence of *B. burgdorferi* infection in patients with cutaneous manifestations suggestive of Lyme disease in Mexico.

## The Study

From June 1999 to October 2000, 4 patients in Mexico City had clinical manifestations suggestive of Lyme disease (*5*,*6*). Two (36 and 54 years of age) had erythema migrans lesions, and 2 (9 and 34 years of age) had borrelial lymphocytoma lesions. Two reported having been bitten by a hard tick; the other 2, by a nonflying insect. Bites occurred while camping in forests: 3 near Mexico City (National Park La Marquesa) and 1 in Quintana Roo, a southern state in Mexico. All patients lived in Mexico City and had never traveled outside Mexico.

Two patients were treated for acute skin lesions (consistent with erythema migrans), malaise, and arthralgia. The skin lesion was an erythematous macula with regular, reddish edges and a pink center. One patient had a 5-cm lesion on the left forearm; the other had a 6-cm lesion on the left thigh. For the 2 other patients, a nodular erythematous cutaneous lesion (consistent with lymphocytoma), 0.5–2 cm in diameter with regular edges, developed 2 months after the bite. One patient’s lesion was on the earlobe; the other’s, on the left cheek.

Serum from each patient was tested for immunoglobulin M (IgM) and IgG against *B. burgdorferi* sensu lato by using a commercial ELISA (cutoff optical density 0.200 and indeterminate 0.200–0.400) (Enzygnost Borreliosis, Dade Behring, Marburg, Germany) (*7*). A *Treponema pallidum* ELISA (Abbott Murex, Wiesbaden, Germany) was performed to rule out cross-reaction with *T. pallidum* infection. Serum samples positive for *B. burgdorferi* by ELISA were further tested by Western blot (WB) by using the Marxblot assay (MarDx Diagnostics, Carlsbad, CA, USA) and Centers for Disease Control and Prevention (CDC) criteria (*5*).

**Figure 1:**
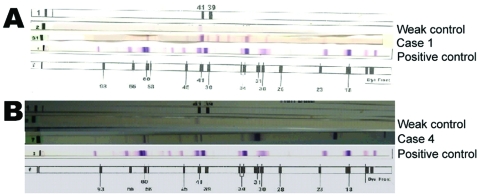
Western Blot (WB) immunoglobulin G results from cutaneous cases. A) WB with serum from patient 1, showing erythema migrans (arrow). B) WB with serum from patient 4, showing lymphocytoma (arrow); a strong positive and a weak positive control were included.

Serum samples from the 2 lymphocytoma patients were positive for *B. burgdorferi* by ELISA and WB (Figure 1, panel A; Table). For the 2 erythema migrans patients, serum samples taken 2 weeks after the tick bite were negative for *B. burgdorferi* IgM and IgG; but 2 months later, 1 patient became seropositive, confirmed by WB (Figure 1, panel B; Table).

**Table:** Serologic IgG and molecular test results for patients with cutaneous lesions suggestive of Lyme borreliosis*

Diagnosis	ELISA OD	WB, molecular weight, kDa†	PCR for *fla* gene	SB‡
Erythema migrans	0.469	23,30,39,41,45,58	+	*Borrelia burgdorferi* sensu stricto
Erythema migrans	0.146	NT	+	*B. burgdorferi* sensu stricto
Lymphocytoma	0.472	23,28,39,41,58,66	+	*B. burgdorferi* sensu stricto
Lymphocytoma	0.574	23*,*39,41,45,66,93	+	*B. burgdorferi* sensu stricto

*Ig, immunoglobulin; OD, optical density; WB, Western blot; SB, Southern blot; NT, not tested. †WB assay with *B. burgdorferi* sensu stricto antigen (Marxblot test), protein size of the bands recognized by the patient`s serum. ‡SB assay was done by using the probe specific for *B. burgdorferi* sensu stricto.

**Figure 2:**
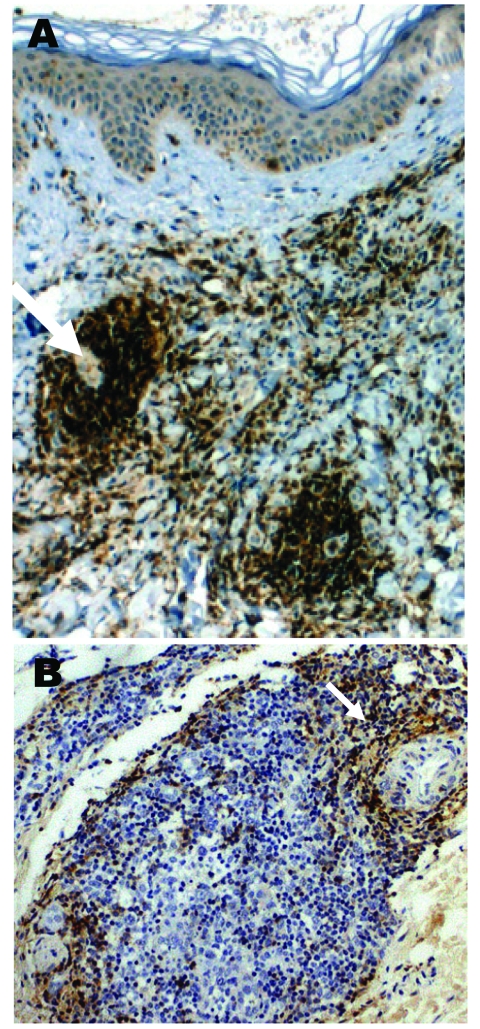
A case of lymphocytoma. A) Immunohistochemical image with anti-CD20 antibody showing a nodule with a dense B-lymphocytes infiltrate in the dermis; magnification ×100. B) Immunohistochemical image with anti-CD45 Ro antibody showing T-lymphocytes at the periphery of a nodule; magnification ×250.

Histologic examination of skin biopsy specimens from each erythema migrans lesion showed a mononuclear cell infiltrate in the superficial and deep dermis; infiltrate included lymphocytes and plasma cells around the perivascular zones. Biopsy samples of lymphocytoma lesions showed dense nodular lymphocytic infiltrates in the reticular dermis with well-delineated lymphoid follicles, no atypical mitosis, B-lymphocytes (anti-CD20, DAKO, Carpentería, CA, USA) in the germinal center (Figure 2, panel A), T-lymphocytes (anti-CD45 RO+) in the follicular zone (Figure 2, panel B), and no CD3+ cells.

DNA was extracted from the biopsy samples (Repli-g Mini Kit, QIAGEN, Valencia, CA, USA) and used for PCR amplification of a fragment of *fla* gene specific for *B. burgdorferi* sensu lato species as well as for a fragment of *ospA* gene, as described (*8*–*10*). DNA from a skin biopsy of a patient with systemic lupus erythematous was used as negative control, and DNA (10 pg/µL) from *B. burgdorferi* sensu stricto B31 served as positive control. All procedures from DNA extraction to amplification were performed twice for each sample. Amplified products were further tested by Southern blot (SB) hybridization with probes specific for *B. burgdorferi* sensu stricto, *B. garinii,* and *B. afzelii*, as described (*9*). DNA from the 4 biopsy samples was positive for *B. burgdorferi* sensu lato *fla* gene by PCR and confirmed as *B. burgdorferi* sensu stricto by SB (Appendix Figure; Table). All DNA biopsy samples were negative by SB with the probes specific for *B. garinii* and *B. afzelii*. We were able to amplify the *OspA* gene for only 1 case of erythema migrans, by using PCR and SB tests (data not shown).

The PCR products of the *fla* gene from 3 patients and of the *ospA* gene from 1 patient were sequenced by using a commercial kit (GenomeLab DTCS-Quick Start Kit, Beckman Coulter, Inc., Fullerton, CA, USA) with the sequencer from Beckman Coulter, Inc., according to manufacturer’s instructions. We used the DNAMAN program (Lynnon Corporation, Vaudreuil-Dorion, Quebec, Canada) to align the sequences with the reported sequences of the *B. burgdorferi* sensu stricto B31 strain (Appendix Figure). For the 2 erythema migrans cases, we found 3 base substitutions (Appendix Figure, panel C), 1 of which was not conserved, leading to a change in amino acid (G for R in the 75 aa); these 2 sequences had 99% homology with the sequence of *B. burgdorferi* *fla* gene of isolate B31 (BLAST program) (*11*). For the lymphocytoma case, we found 2 base substitutions, the same as those of the erythema migrans cases, including the nonconserved base substitution (Appendix Figure, panel C).

Regarding the *ospA* gene in the erythema migrans case, the sequence showed 1 base substitution that was not conserved, leading to a change in the amino acid 5 (L for I). The sequence of this case had 99% homology with the plasmid Ip54 gene of B31strain sequence (*11*) (Appendix Figure, panel D).

The 3 adult patients received doxycycline 200 mg/day for 3 weeks; the child received amoxicillin 50 mg/kg a day for 3 weeks. For all patients, lesions were gone at the end of the treatment and had not recurred 3 years later.

## Conclusions

Erythema migrans is the diagnostic marker for Lyme disease associated with *B. burgdorferi* infection (*5*,*6*). Histologic data from our 2 erythema migrans cases agreed with data reported for other erythema migrans cases (*5*). Moreover, the 2 erythema migrans cases were positive for *B. burgdorferi* sensu stricto *fla* gene and 1 for *ospA* gene; the 3 cases had a high degree of homology to the sequences of strain B31. In addition, 1 case met CDC criteria for seropositivity to *B. burgdorferi* infection (*5*).

Borrelial lymphocytoma is a rare clinical entity reported mostly in Europe (*12*–*14*) and sporadically in the United States (*15*). In this study, histologic and immunohistochemical data from the 2 lymphocytoma cases agreed with data from previous cases. These results were not specific enough to be considered diagnostic; however, germinal centers are present in 80% of borrelial lymphocytoma cases (*12*). Serum samples from 2 patients were positive by WB, which fulfills CDC criteria (*5*). In 1 case, *fla* gene was amplified and sequenced, showing high homology with the *fla* gene from *B. burgdorferi* sensu stricto strain B31 (*11*). Few reports describe genotyping of *B. burgdorferi* species in borrelial lymphocytoma. In Slovenia, *B. afzelii* and *B. bissettii* were identified (*13*); in Germany, *B. garinii* was identified (*14*). In our lymphocytoma patients, we identified *B. burgdorferi* sensu stricto. That the 2 borrelial lymphocytoma cases occurred in patients who had visited the same national park suggests that *B. burgdorferi* is endemic to that area.

This study documents *B. burgdorferi* infection in Mexican patients. Relevant epidemiologic data are 1) cases occurred after visiting forest areas, 2) patients reported having been bitten by a nonflying insect, 3) cases occurred during the summer-fall season, 4) no patient reported having traveled to another country, and 5) all skin lesions resolved after treatment with an antimicrobial drug. Our results suggest that *B. burgdorferi* infection occurs in Mexico and that continuous surveillance for Lyme disease in Mexico should be mandatory.

## Supplementary Material

Appendix Figure Molecular evidence for Borrelia infection. A) PCR for fla gene from Borrelia burgdorferi sensu lato; B) Southern blot assay with probes specific for B. burgdorferi sensu stricto; C) Sequences of fla gene amplified from 2 patients with erythema migrans (lines 1 and 2) and 1 with lymphocytoma (line 3) and aligned with the sequence of the fla gene from B. burgdorferi sensu stricto strain B31;and D) Sequence of the osp A gene amplified from a patient with erythema migrans (line 1) and aligned with the ospA gene from B. burgdorferi sensu stricto strain B31.
