# Supplementary figures and images for: Borrelia burgdorferi Infection and Cutaneous Lyme Disease, Mexico
Source: Emerg Infect Dis. 2007 Oct;13(10):1556–8. doi: 10.3201/eid1310.060630 (PMC2851501; doi:10.3201/eid1310.060630)

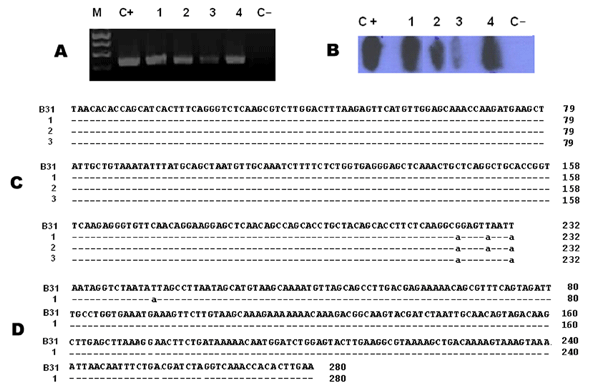

Supplement: Appendix Figure — Molecular evidence for Borrelia infection. A) PCR for fla gene from Borrelia burgdorferi sensu lato; B) Southern blot assay with probes specific for B. burgdorferi sensu stricto; C) Sequences of fla gene amplified from 2 patients with erythema migrans (lines 1 and 2) and 1 with lymphocytoma (line 3) and aligned with the sequence of the fla gene from B. burgdorferi sensu stricto strain B31;and D) Sequence of the osp A gene amplified from a patient with erythema migrans (line 1) and aligned with the ospA gene from B. burgdorferi sensu stricto strain B31. [file 06-0630_appF-s1.gif]
